# The influence of common method bias on the relationship of the socio-ecological model in predicting physical activity behavior

**DOI:** 10.15171/hpp.2018.05

**Published:** 2018-01-07

**Authors:** Savanna Wingate, Eveleen Sng, Paul D. Loprinzi

**Affiliations:** ^1^Physical Activity Epidemiology Laboratory, Exercise Psychology Laboratory, Department of Health, Exercise Science and Recreation Management, The University of Mississippi, University, MS 38677, USA

**Keywords:** Exercise psychology, Inflation, Self-efficacy

## Abstract

**Background:** The purpose of this study was to evaluate the extent, if any, that the association between socio-ecological parameters and physical activity may be influenced by common method bias (CMB).

**Methods:** This study took place between February and May of 2017 at a Southeastern University in the United States. A randomized controlled experiment was employed among 119 young adults.Participants were randomized into either group 1 (the group we attempted to minimize CMB)or group 2 (control group). In group 1, CMB was minimized via various procedural remedies,such as separating the measurement of predictor and criterion variables by introducing a time lag (temporal; 2 visits several days apart), creating a cover story (psychological), and approximating measures to have data collected in different media (computer-based vs. paper and pencil) and different locations to control method variance when collecting self-report measures from the same source. Socio-ecological parameters (self-efficacy; friend support; family support)and physical activity were self-reported.

**Results:** Exercise self-efficacy was significantly associated with physical activity. This association (β = 0.74, 95% CI: 0.33-1.1; P = 0.001) was only observed in group 2 (control), but not in group 1 (experimental group) (β = 0.03; 95% CI: -0.57-0.63; P = 0.91). The difference in these coefficients (i.e., β = 0.74 vs. β = 0.03) was statistically significant (P = 0.04).

**Conclusion:** Future research in this field, when feasible, may wish to consider employing procedural and statistical remedies to minimize CMB.

## Introduction

Self-report measurement of physical activity is most commonly used in physical activity literature because it is low cost, feasible, convenient and easy for researchers to administer.^1^ The socio-ecological model provides a comprehensive framework for understanding the multiple levels of influence (e.g., intrapersonal and interpersonal) on physical activity behavior.^2^ For example, self-efficacy and social support have been shown to have positive influences on physical activity.^3-6^ However, most of these aforementioned constructs are usually measured by self-report measures and common method bias (CMB) could, potentially, inflate relationships between variables in behavioral research.^7^ The topic of CMB, or shared method variance, has been of great interest recently.^8-10^

Podsakoff and colleagues^7^ identified a list of potential sources of CMB, which includes, for example, mood state and social desirability^11-13^ of participants when completing surveys, length of surveys, and measurement context effects (i.e., predictor and criterion variables measured at the same point in time, same location, and same medium). They also provided potential procedural remedies to consider, such as separating the measurement of predictor and criterion variables by introducing a time lag (temporal), creating a cover story (psychological), and proximally separating measures to have data collected in different media (computer-based vs. paper and pencil) and different locations to control method variance when collecting self-report measures from the same source.

As noted, Podsakoff et al^7^ has comprehensively detailed the potential sources and implications of CMB. To our knowledge, however, no experimental study has applied the procedural remedies (e.g., temporal, psychological, different media) recommended by Podsakoff et al within the domain of physical activity. Thus, using the socio-ecological framework, the purpose of this study was to employ an experimental study to evaluate the extent, if any, that the association between socio-ecological parameters and physical activity may be influenced by CMB.

## Materials and Methods

### Study design and participants

This study took place between February and May of 2017 at a Southeastern University in the United States. A total of 130 participants (students at the authors’ institution) were recruited via a convenience sampling approach. This sample size was based on our previous pilot work (unpublished) on this topic. They were eligible for the study if they were aged 18 and over, and consent was implied if they completed the questionnaires during their lab visit. After careful review of the data, 11 persons were excluded from the analysis because of missing data. The final number of participants was 119. There were no differences (*P* > 0.05) in the study variables between the 119 participants and the 11 excluded participants.

The 119 participants had a mean age of 21.2 (±1.8) years. The majority of participants identified as Caucasian (82.4%), African American (10.1%), and other (7.5%). Regarding gender, 34.5% identified themselves as males.

In this parallel-group, randomized controlled experimental design, there were 2 experimental arms. Participants were randomized, via a computer-generated list, into either group 1 (the group we attempted to minimize “common method bias”; n = 56) or group 2 (the control group; n = 63). Details are explained below.

### Surveys

Participants completed a demographic survey, Physical Activity Vital Sign (PAVS; to assess physical activity behavior),^14^ Exercise Self-Efficacy,^15^ Social Support for Exercise,^16^ and the Positive and Negative Affect Schedule (PANAS).^17^ Group 1 completed an additional Starting the Conversation Questionnaire^18^ and self-reported their height and weight. Justification for these assessments is described below.

With regard to the outcome variable, the PAVS is a brief survey tool, composed of 2 questions. (1) “How many days per week, on average do you engage in moderate to vigorous intensity physical activity (including a brisk walk),” and (2) “How many minutes, on average do you engage in this physical activity?” The product of these 2 variables was used to calculate weekly engagement in physical activity. This simple questionnaire has been shown to exhibit adequate reliability,^14^ and is correlated with accelerometer-assessed number of days ≥30 min/d of moderate-to-vigorous physical activity (*r* = 0.52, *P* < 0.001).^19^

With regard to the 2 main independent variables (self-efficacy and social support), self-efficacy was assessed from a 6-item scale assessing exercise self-efficacy. For each item, response options ranged from 0-100. An example item is, “I am able to exercise 3 times per week at a moderate intensity for 40+ minutes without quitting for the next month (0-100; not at all confident to highly confident).” For group 1 and group 2, respectively, internal consistency, as measured by Cronbach’s alpha, was 0.978 and 0.979.

Regarding social support, family and friend social support were assessed. For both family and friend social support, 13 items were used, ranging from 1-5 (none to very often). An example item is, “During the past three months, my [family or friend] gave me encouragement to stick with my exercise program.” For group 1 and group 2, respectively, internal consistency, as measured by Cronbach’s alpha, was 0.81 and 0.86 for family support. For group 1 and group 2, respectively, internal consistency, as measured by Cronbach’s alpha, was 0.87 and 0.85 for friend support.

### Procedure

Participants randomized into group 1 (n = 56) completed 2 visits to our 2 different laboratories. For group 1 we attempted to minimize CMB through recommendations from Podsakoff et al.^7^ For their first visit, they visited the Exercise Psychology Laboratory and completed surveys assessing the socio-ecological predictor (psychosocial) variables in a paper-and-pencil format. During that visit, they were told that the purpose was to evaluate the association between the socio-ecological parameters and dietary behaviors, which was specifically used as our cover story. Within approximately 1-3 days, participants came back for testing, but completed a survey on the criterion (physical activity) variable online via Qualtrics in the Physical Activity Epidemiology Laboratory. They were told that the purpose of visit 2 was to look at the relationship between perceived weight status and physical activity, which again, was used as a cover story.

The above protocol was conducted in this manner to separate the measurement of the predictor and criterion variables by introducing a time lag (temporal), creating a cover story (psychological), and proximally separating measures to have data collected in different media (computer-based vs. paper and pencil) and different locations; this was done to (attempt to) control for potential common method variance when collecting self-report measures from the same source. Notably, for all questionnaires in visit 1 and visit 2, the headings and scoring information were removed from the original survey. Also, participants in group 1 were told to not write their name on any of the surveys to ensure anonymity and to answer as honestly and accurately as they could. Participants in group 1 answered the PANAS questionnaire at the start of both visits to account for their mood state.

Participants randomized into group 2 (n = 63; i.e., the “control group”) completed a single visit in the Exercise Psychology Laboratory. During this visit, they completed all the questionnaires pertaining to the predictor (socio-ecological parameters) variables and criterion variable (physical activity) in a paper-and-pencil format, including the PANAS scale. The questionnaires were presented in the original formats (i.e., section headers were displayed on surveys); participants were informed of the actual purpose of the study (to evaluate the association between socio-ecological parameters and physical activity; thus, a “cover story” was not employed) and they were also told to write their name on the demographic questionnaire.

### Statistical analysis

The data were analyzed using Stata (v.14; College Station, TX, USA). Independent samples *t* tests (for continuous variables) and chi-square analyses (for categorical variables) were used to evaluate demographic, behavioral and socio-ecological differences between the 2 groups. Multiple linear regression analysis was used to examine the associations between the socio-ecological parameters and physical activity. Assumptions of linear regression (e.g., normality, non-collinearity) were checked and confirmed to not be violated. Models were computed separately for each group, and for each group, the outcome variable was physical activity and the independent variables were the socio-ecological parameters. Statistical significance was set at a nominal alpha of 0.05.

## Results

Characteristics of the study variables, stratified by group allocation, are shown in Table 1. There were no significant differences for any of the variables between the 2 groups (all *P*’s > 0.05).

The multivariable linear regression analyses are shown in Table 2. Exercise self-efficacy was the only variable that was significantly associated with physical activity. Notably, this association (β=0.74, 95% CI: 0.33-1.1; *P* = 0.001) was only observed in group 2 (the group that we did not attempt to minimize CMB), but not in group 1 (experimental group) (β = 0.03; 95% CI: -0.57-0.63; *P* = 0.91). Importantly, the difference in these coefficients (i.e., β = 0.74 vs. β = 0.03) was statistically significant (*P* = 0.04). Additionally, results were unchanged when including the mood (PANAS) assessment as a covariate in the models (data not shown). Lastly, in all models, the highest variance inflation factor was 1.2, demonstrating that the observed differences between the groups was not a result of differences in mood state or multicollinearity. Further, internal consistency of the socio-ecological variables was reasonable for each group, further demonstrating that the observed group difference in self-efficacy was not a result of a differential degree of reliability (internal consistency).

## Discussion

The purpose of this study was to use the socio-ecological framework to evaluate the extent, if any, that the association between socio-ecological parameters and physical activity is influenced by CMB. Our results demonstrate some potential evidence of CMB. That is, exercise self-efficacy was significantly associated with physical activity, but this association was statistically significant only in the group that we did not attempt to control for CMB.

Regarding self-efficacy being the only significant socio-ecological parameter, this finding is consistent with findings from other studies showing that self-efficacy is one of the stronger socio-ecological correlates of physical activity.^3,20,21^ The study sample and environment may be another potential explanation for why self-efficacy was the only significant socio-ecological parameter. For example, the present sample was highly active, which may have augmented the association between self-efficacy and physical activity. Further, all participants lived within the same college town, and as such, this homogeneity in environment may have attenuated (due to limited variability) potential associations between the other evaluated socio-ecological parameter (support) on physical activity.

In conclusion, and based on our findings, we wish to suggest that CMB may, in part, potentially influence the relationship between socio-ecological parameters (particularly self-efficacy) and physical activity. If our findings are replicated by future research, then, when feasible, physical activity investigators should consider employing procedural and statistical remedies to minimize CMB. A major strength of our study is its novelty and experimental study design. However, a limitation of this study is the homogenous sample (college students), and thus, the results may not be generalizable to other populations. The relatively small sample size is also a limitation of the experimental study. Given the potential temporal fluctuations in the measures, it is also possible that the time separation introduced in the experimental group between the two sets of measures might have introduced differential results in a manner that is not related to the use of shared method variance. However, we believe that this is unlikely given that some of the measures assessed typical (e.g., week long) levels of the behavior, as opposed to a single day behavior assessment. Further, these measures, including self-efficacy (which may have greater daily fluctuations) have demonstrated evidence of test-retest reliability. Future recommendations include replicating this study in other populations, and implementing an objective measure of physical activity. This latter point will help us to further determine if CMB does, indeed, inflate the associations between the socio-ecological parameters and physical activity. For example, if a future study replicates our results (e.g., self-efficacy is only associated with self-reported physical activity in the “control group”), and then also demonstrates that the association between the socio-ecological parameters and *objectively-measured* physical activity is similar across the 2 intervention groups, then this will provide more concrete evidence to suggest that CMB may, in part, inflate the relationship between the socio-ecological framework and self-reported physical activity. Until such a study is conducted, it is still uncertain as to the extent, if any, that CMB may be influencing the relationship between socio-ecological parameters and physical activity.

## Ethical approval

All procedures performed in studies involving human participants were in accordance with the ethical standards of the institutional and/or national research committee and with the 1964 Helsinki Declaration and its later amendments or comparable ethical standards. Informed consent was not obtained as this study was IRB exempt given the survey-based nature of this study. This study was approved by the Institutional Review Board at the authors’ institution (Protocol 17x-151).

## Competing interests

SW, ES and PL declare no conflict of interest.

## Authors’ contributions

ES and PL performed analyses. SW collected the data. All authors have reviewed and edited the manuscript. All authors have read and approved the final version of the manuscript, and agree with the order of presentation of the authors.

**Table 1:** Characteristics of participants (mean/proportion [SD])

**Measure**	**Overall sample (N = 119)**	**Group 1 (n = 56)**	**Group 2 (n = 63)**	***P*** **value** ^a^
Age, mean years	21.2 (1.8)	21.3 (2.1)	21.2 (1.4)	0.58
Sex, % male	34.5	34.0	35	0.91
Race, % white	82.4	84.0	81.0	0.62
Exercise Self-Efficacy, mean sum	456.7 (140.1)	440.1 (133.9)	471.4 (144.8)	0.22
Social Support, mean				
Family	26.4 (11.4)	25.7 (9.9)	27.1 (12.7)	0.53
Friends	30.8 (9.6)	31.3 (8.3)	30.4 (10.7)	0.61
Physical Activity, mean min/wk	238.9 (273.3)	225.0 (253.2)	251.2 (223.6)	0.55
PANAS, mean sum				
Positive – Visit 1	30.4 (7.3)	29.9 (6.4)	30.8 (8.0)	0.50
Negative – Visit 1	13.8 (4.0)	13.5 (3.2)	14.0 (4.6)	0.54
Positive – Visit 2		28.6 (7.0)		
Negative – Visit 2		13.8 (4.5)		

^a^ *P* value examines differences between group 1 and group 2. An independent samples *t* test was used for continuous variables and a chi-square analysis was used for categorical variables. Group 1, the group we attempted to minimize CMB; Group 2, control group.

**Table 2:** Regression model examining the associations between predictor and criterion variables

**Measure**	**Group 1 (n = 56)**			**Group 2 (n = 63)**		
	**β**	***P *** **value**	**95% CI**	**β**	***P *** **value**	**95% CI**
Exercise Self-Efficacy	0.03	0.91	-0.57, 0.63	0.74	0.001	0.33, 1.1
Social Support						
Family	0.45	0.90	-7.4, 8.3	-3.7	0.25	-10.2, 2.7
Friends	3.3	0.44	-5.2, 11.9	3.8	0.28	-3.3, 10.9

Two separate regression models were computed, with physical activity as the outcome variable and the socio-ecological variables as the independent variables. One model was computed for group 1 (minimized CMB group) and another was computed for group 2 (control group).
